# Chronic pain-related cortical neural activity in patients with complex regional pain syndrome

**DOI:** 10.1016/j.ibneur.2021.05.001

**Published:** 2021-05-13

**Authors:** Katsuyuki Iwatsuki, Minoru Hoshiyama, Akihito Yoshida, Jun-ichi Uemura, Aiko Hoshino, Izumi Morikawa, Yasunobu Nakagawa, Hitoshi Hirata

**Affiliations:** a Department of Hand Surgery, Graduate School of Medicine, Nagoya University, 65 Tsurumai-cho, Showa-ku, Nagoya, Aichi 466-8550, Japan; b Department of Health Sciences, Faculty of Medicine, Nagoya University, 1–1-20 Daiko-minami, Higashi-ku, Nagoya, Aichi 461-8673, Japan

**Keywords:** Complex regional pain syndrome (CRPS), Chronic pain, Magnetoencephalography (MEG), Connectivity, Default mode network (DMN), Amplitude envelope correlation (AEC)

## Abstract

Quantitative objective measurement of chronic pain is important. We elucidated chronic pain-related cortical neural activity and neural connectivity among pain-related brain regions in complex regional pain syndrome (CRPS). Resting-state magnetoencephalography recordings were performed. Cortical current density and neural connectivity, revealed by amplitude envelope correlation (AEC), were estimated on standardized brain magnetic resonance imaging. Intra-experiment pain was assessed subjectively using a visual analogue scale (VAS). The correlation between current density and VAS scores was calculated for the occipital areas and pain-related cortices. Current density in the primary (SI) and secondary (SII) somatosensory cortex and precuneus in both hemispheres was negatively correlated with the pain VAS score. The AEC and VAS values were significantly correlated for the SII and the precuneus and for the SII and insular cortex in the alpha frequency band in the right hemisphere. In the theta frequency band, the AEC and VAS values correlated for the SII and posterior cingulate cortex in the right hemisphere. Our results suggested that disruption of pain processes and functions in the default mode network occurs in CRPS. Our method targeting the neural mechanism of pain has the potential to offer a clinically objective means of evaluating it.

## Highlights

• Chronic pain can be objectively evaluated with magnetoencephalography.
• Neural connectivity of pain-related cortices was reduced in complex regional pain syndrome.
• Default mode network is disrupted in complex regional pain syndrome patients.

## Nomenclature

(ACC): anterior cingulate cortex
(AEC): amplitude envelope correlation
(CRPS): complex regional pain syndrome
(DMN): default mode network
(FDR): false discovery ratio
(LO): lateral occipital area
(MRI): magnetic resonance imaging
(MEG): magnetoencephalography
(PCC): posterior cingulate cortex
(SI): primary somatosensory cortex
(SII): secondary somatosensory cortex
(VAS): visual analogue scale

## 1 Introduction

Chronic pain has maladaptive effects on the quality of life, capacity for work and ability to function within society of those afflicted by it (Yoshida et al., 2019); further, the effects of chronic pain strain public, private and personal resources. In the clinical environment, it is typically evaluated subjectively such as by eliciting behavioral responses or by administering a visual analogue scale (VAS) or numerical rating scale (Langley and Sheppeard, 1985, Skovlund and Breivik, 2016). But these methods have limited reliability and present challenges for clinicians, such as in the detection of malingering in a traffic or industrial accident compensation claim. The objective, quantitative measurement of chronic pain is salient in the clinical as well as basic neurosciences. However, the pathophysiology of chronic pain remains unknown, although many studies have been performed from the molecular to system levels (Sommer, 2016).

Central nervous system sensitization may influence pain chronicity. Recent studies have investigated chronic pain mainly by means of functional magnetic resonance imaging (fMRI) (Cagnie et al., 2014, Shokouhi et al., 2018). Although a very useful means to evaluate chronic pain, fMRI has shortcomings, including the inability to evaluate some patients (Iwatsuki et al., 2020). Changes in blood flow have been measured by fMRI, but changes in neural activity that do not result in an increase in oxygen consumption cannot be elucidated by fMRI (Laufs et al., 2006). On the other hand, magnetoencephalography (MEG) is capable of detecting neural activity in the brain with high temporal resolution and reliability in electrical source estimation (Hari and Salmelin, 2012, Iwatsuki et al., 2019a).

Complex regional pain syndrome (CRPS) is a refractory, severe type of chronic pain. It presents with characteristic symptoms of chronic pain, although the degree of pain is disproportionately intense. Many CRPS patients have difficulty in daily life due to pain, which can have socio-economic consequences. Elucidation of the pathophysiology and establishment of novel treatments for CRPS are needed. The existing diagnostic criteria for CRPS are suitable to identify patients for targeted intervention and research (Harden et al., 2007, Sumitani et al., 2010).

If a method to target and analyze CRPS-specific brain function is identified, it could allow us to objectively evaluate the condition and even clarify its diagnosis. Additionally, such an analytical method could be applicable to other types of chronic pain in the future. Here, we conducted brain function analysis in patients with CRPS using MEG to clarify its etiology and pathology.

## 2 Experimental procedures

### 2.1 Participants

Outpatients with CRPS were recruited at the Department of Hand Surgery, Nagoya University. Diagnostic criteria were based on the “Comprehensive Diagnostic Criteria for Complex Regional Pain Syndrome in the Japanese Population” (Sumitani et al., 2010). Patients with a clinical history of CRPS having scores of 3 or more in both objective and subjective assessments of trophic change, decreased range of motion, abnormal pain processing, sudomotor activity, and edema were included. Since the present study entailed measurements via MEG and MRI, participants with claustrophobia were excluded. Control participants were healthy volunteers with no previous history of neurological diseases and no symptoms of subjective pain.

Written informed consent was obtained from each participant prior to the study. The study was approved by the Ethics Committee of the Nagoya University Graduate School of Medicine (2015–0081) and was conducted in accordance with the Declaration of Helsinki.

### 2.2 Experimental setting

Participant’s MEG signals at rest were recorded and their subjective pain was assessed by VAS at each time-point. The MEG and subjective pain measurements were recorded two or three times in patients with CRPS at an interval of at least 6 months.

### 2.3 MEG recording

The MEG signals were recorded in a magnetically shielded room with a whole-head MEG system (PQ-1160C, Ricoh Co., Tokyo, Japan). The MEG system included 160-channel, axial-type, first-order gradiometers with a 50-mm-long baseline detection coil. The gradiometers were arranged in a uniformly distributed array on a helmet-type dewar. Participants were asked to lie on their back with their head in the MEG dewar, remaining awake but resting with eyes closed for 4 min while MEG signals were recorded. The MEG and electrocardiogram signals were recorded with an initial bandpass filter of 0.3–2000 Hz and a notch filter of 60 Hz at a sampling rate of 5000 Hz (Iwatsuki et al., 2016, Iwatsuki et al., 2019b).

### 2.4 Recording of fiducial points and brain imaging recordings

Prior to MEG recording, the fiducial points, bilateral pre-auricles, nasion, three points on the forehead (6 cm above nasion on the midline [MF] and points 5 cm lateral to the right and left from the MF), and a tracing of the scalp surface were used to obtain the Montreal Neurological Institute (MNI) stereotactic coordinates for each participant. The origin of the coordinate system was the midpoint between the pre-auricular points. The x-axis joined the origin to the nasion such that the positive value oriented towards the nasion. The positive y-axis extended from the origin through the left side, and the positive z-axis extended from the origin through the vertex.

Participants underwent MRI at the Nagoya University's Brain and Mind Research Center using a Siemens Magnetom Verio (Siemens, Erlangen, Germany) 3.0-T scanner with a 32-channel head coil. T1-weighted MR images were acquired using a 3D Magnetization Prepared Rapid Acquisition Gradient Echo (MPRAGE, Siemens) (Mugler and Brookeman, 1990) pulse sequence from all participants with the following imaging parameters: repetition time/MPRAGE repetition time = 7.4/2500 ms, echo time = 2.48 ms, inversion time = 900 ms, 192 sagittal slices with a distance factor of 50% and 1-mm thickness, field-of-view = 256 mm, 256 × 256 matrix dimension, in-plane voxel resolution of 1.0 × 1.0 mm^2^, flip angle = 8 degrees, and total scan time = 5 min 49 s.

Individual 3D MR images were transferred to a pseudo-individual anatomy by BrainSuite software (Shattuck and Leahy, 2002). The pseudo-individual anatomy was the standard brain anatomy, from the International Consortium for Brain Mapping’s (ICBM) 152 non-linear atlases (Fonov et al., 2009) that were created using BrainStorm software (Tadel et al., 2011). The number of vertices of the cortical surface of the pseudo-individual brain was 7501 in the present study. Further anatomical and MEG signal analyses were performed for this brain model. When MR images could not be obtained from a participant due to metals around the brain or any other reason, the pseudo-individual brain anatomy was obtained from fiducial points and the surface of the scalp by BrainStorm software.

### 2.5 Evaluation of subjective pain

Subjective pain at the time of the experiment was evaluated by a VAS ranging from 0 (no pain) to 100 (strongest pain imaginable). The patient was asked to place a mark on a horizontal line, 100 mm in length, to indicate the level of intensity of his or her pain (Langley and Sheppeard, 1985, Skovlund and Breivik, 2016).

### 2.6 Data analyses

The MEG signals were downsized for further analysis by a sampling frequency at 1000 Hz with a bandpass filter between 3 and 150 Hz. Prior to the analysis, the electrocardiogram and eye-blink artifacts were removed in the pre-analysis preparation process, using the Signal-Space Projection and Independent Component Analysis methods (Uusitalo and Ilmoniemi, 1997).

#### 2.6.1 Cortical areas activated during chronic pain

**Fig. 1:**
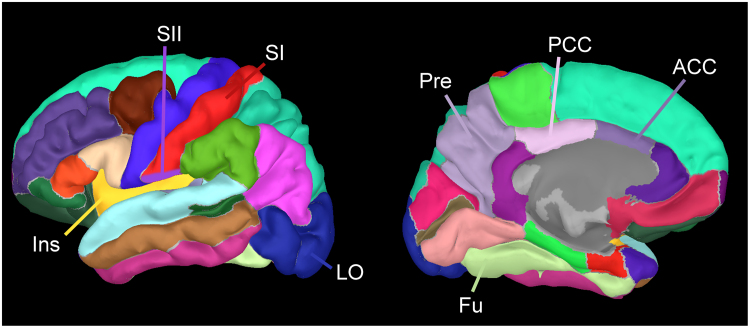
Cortical parcellations based on the Desikan-Killiany parcellations. Regions indicated in the figure were those used in the correlation analyses between the current density and subjective pain, and in the connectivity analyses among pain-related areas. Abbreviations: SI, primary somatosensory cortex; SII, secondary somatosensory cortex; Ins, insular cortex; LO, lateral occipital area; Fu, fusiform gyrus; Pre, precuneus; ACC, anterior cingulate cortex; PCC, posterior cingulate cortex.

Current density at each vertex point on the cortex was estimated by minimum norm estimation (Hämäläinen and Ilmoniemi, 1994). Cortical current density was compared between two groups, participants with no to mild subjective pain (VAS < 30) and those with moderate to severe subjective pain (VAS ≥ 30) at each vertex on the cortex based on the parcellations of Desikan-Killiany (Desikan et al., 2006) (Fig. 1), by a *t*-test using the false discovery ratio (FDR) for multiple comparisons.

#### 2.6.2 Neural connectivity among pain-related cortical areas

A pain matrix of 12 pain-related cortical areas in each side of the brain were selected. Neural connectivity between each pain-related cortical area and the other 11 cortical areas was assessed. Inter-regional functional connectivity between two cortical areas was calculated as the synchrony of the MEG signals, which was expressed by amplitude envelope correlation (AEC) with orthogonalization (Colclough et al., 2016). The AEC value was obtained at each frequency band, i.e. theta (between 4 and 7 Hz), alpha (between 8 and 12 Hz), and beta (between 13 and 30 Hz). Correlations between the AEC and VAS values were calculated by Pearson’s test with FDR for all 51 MEG recordings.

## 3 Results

### 3.1 Participants

Twenty-one patients with CRPS (7 males and 14 females, mean age 51.8 years, age range 18–86 years) were included. The total number of measurements for patients with CRPS was 42. Twenty-one control participants (8 males and 13 females, mean age: 52.6 years, age range: 22–76 years) were involved in the study. 63 MEG recordings were analyzed.

### 3.2 Comparisons of cortical current among pain levels

**Fig. 2:**
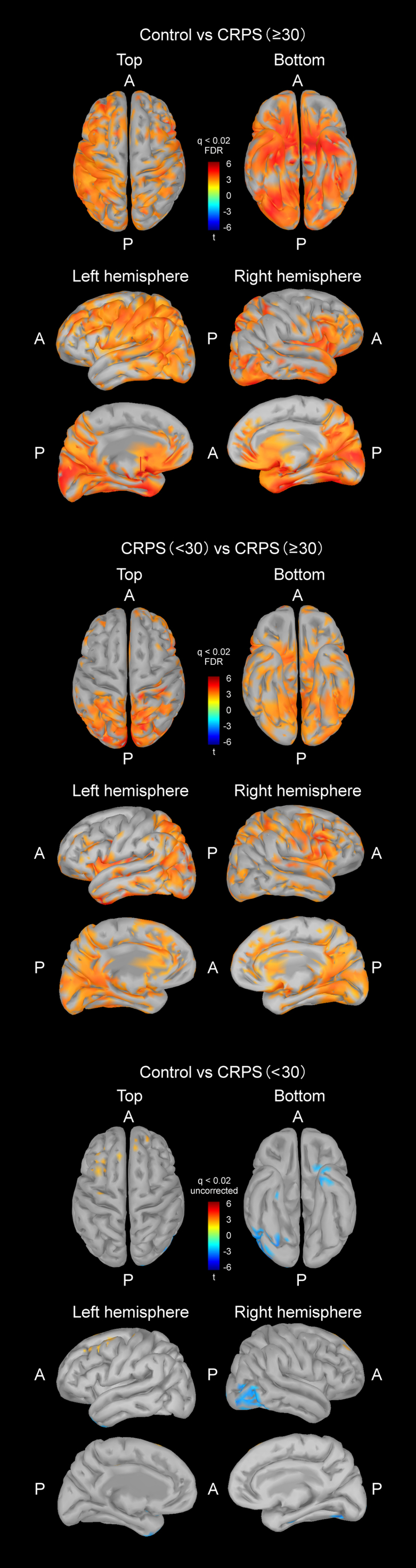
Brain areas with significant differences in current density between patients with weak (VAS score < 30) and strong (VAS score ≥ 30) subjective pain, and between controls and those with strong subjective pain. Occipital, posterior-inferior temporal, primary somatosensory cortex, secondary somatosensory cortex, precuneus, insula and anterior cingulate areas in each hemisphere were included (*t*-test, q < 0.02). Uncorrected comparison showed some areas with differences between the two groups (*t*-test, q < 0.02). Abbreviations: A, anterior; P, posterior.

The number of participants with a pain VAS score <30 and those with a score ≥30 was 18 and 24, respectively. The VAS score of each control participant was 0 (n = 21). The lateral occipital (LO) and fusiform areas as well as the pain matrix (Legrain et al.,2011) (i.e. primary [SI] and secondary [SII] somatosensory cortex, insula, anterior cingulate cortex [ACC], posterior cingulate cortex [PCC], and precuneus regions) had significant differences between the VAS < 30 and VAS ≥ 30 groups. Correlations between current density and VAS scores were assessed in these cortical areas (Pearson’s test with FDR). The cortical areas showing significant differences in current density between these groups are shown in Fig. 2; these were the occipital, posterior-inferior temporal, SI, SII, precuneus, insula, and ACC areas in both hemispheres (q < 0.05, FDR). The value of current density was larger in participants with a pain VAS score <30 than in those with a VAS score ≥30.

### 3.3 Correlation between subjective pain and regional current density

**Fig. 3:**
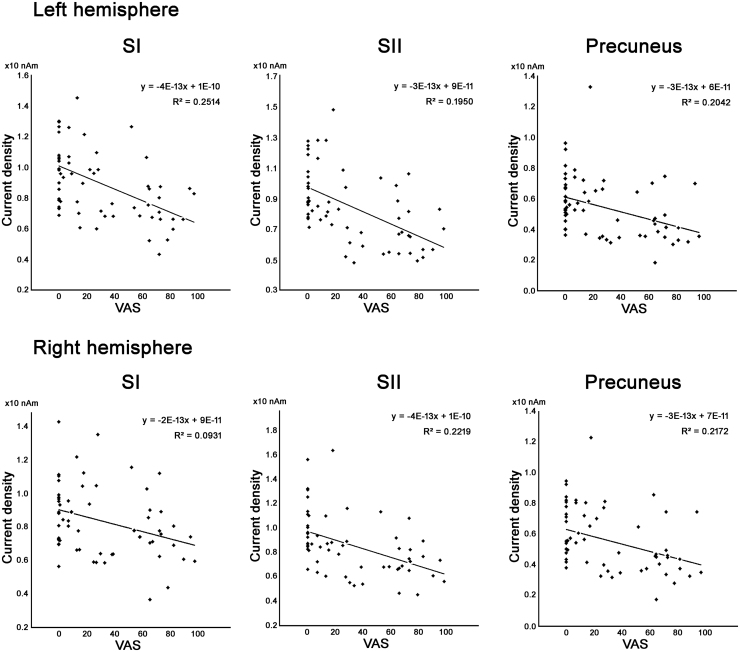
Correlation between regional current density and pain visual analogue scale (VAS) score. Cortical areas with significant correlation listed in Table 1, i.e. SI, SII, and precuneus in both hemispheres, are shown (q < 0.05). Abbreviations: SI, primary somatosensory cortex; SII, secondary somatosensory cortex; VAS, visual analogue scale Solid lines indicate regression curves.

**Fig. 4:**
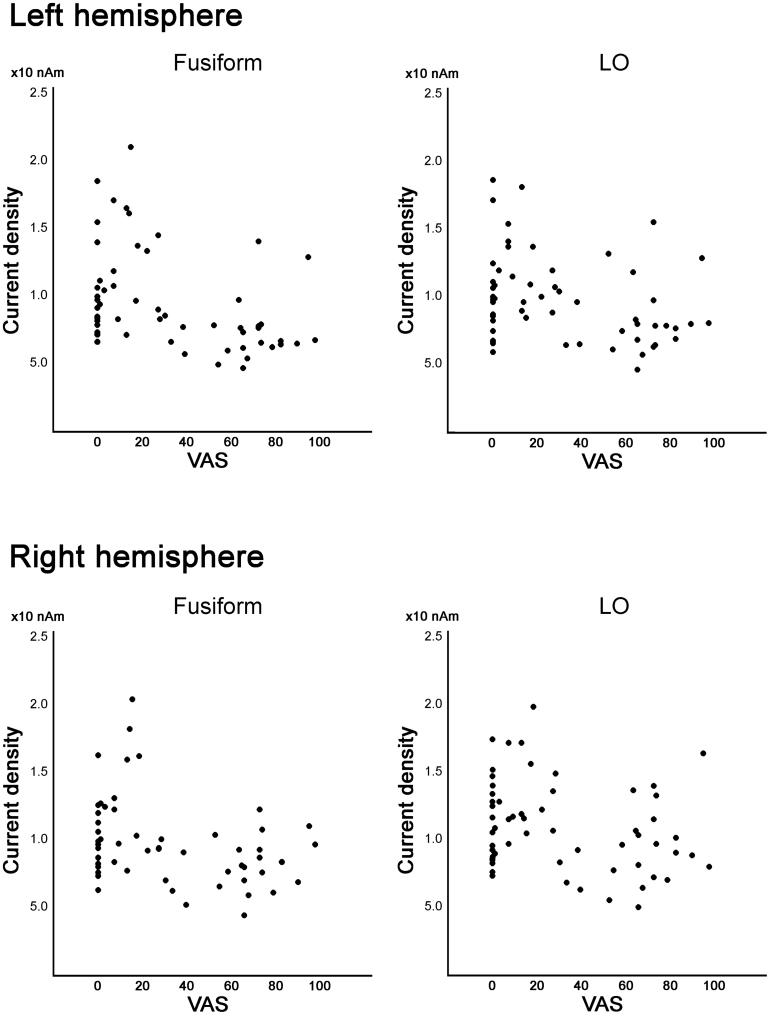
Correlation between regional current density and pain visual analogue scale (VAS) score in the fusiform and lateral occipital (LO) areas. In those areas, the current density was larger in the participants with a VAS score <30 than in those with a VAS score ≥30, but the linear correlation between current density and VAS was not significant.

The correlation between current density and VAS scores was calculated for the occipital areas and pain-related cortices. Current density in SI, SII and precuneus in both hemispheres was negatively correlated with the pain VAS score (q < 0.05, FDR; Table 1 and Fig. 3). In the fusiform and LO, the current density was larger in participants with a VAS score <30, including controls, than in those with a VAS score ≥30. However, the correlation between current density and VAS score was not statistically significant (Fig. 4).

**Table 1:** Correlation between current density and pain VAS in the pain-related cortices and occipito-temporal cortices selected from Fig. 2.

Side	Pain-related and occipito-temporal cortices	Correlation coefficient	q value (FDR)
L	SI	－0.438	0.0080**
R	Precuneus	－0.372	0.0311*
L	SII	－0.367	0.0241*
L	LO	－0.367	0.0568
L	Precuneus	－0.366	0.0148*
R	SI	－0.343	0.0228*
R	SII	－0.214	0.0405
L	Insula	－0.244	0.1524
L	Fusiform	－0.237	0.4755
R	Insula	－0.235	0.1437
L	ACC	－0.231	0.1416
R	ACC	－0.219	0.1589
L	PCC	－0.193	0.2234
R	LO	－0.192	0.6466
R	ACC	－0.182	0.2298
R	Fusiform	－0.176	0.7187

Abbreviations: SI, primary somatosensory cortex; SII, secondary somatosensory cortex; ACC, anterior cingulate cortex; PCC, posterior cingulate cortex; LO, lateral occipital area.

* q < 0.05.

** q < 0.01.

### 3.4 Correlation between pain and neural connectivity

**Fig. 5:**
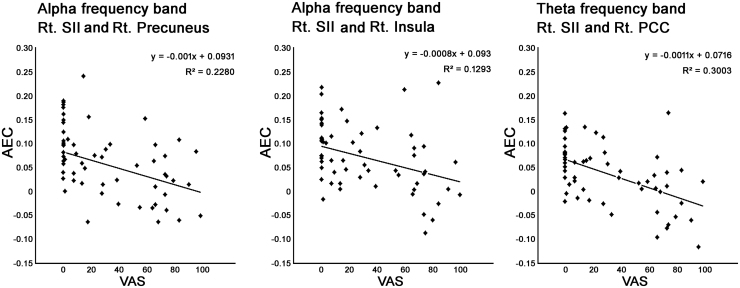
Correlation between amplitude envelope correlation (AEC) and pain visual analogue scale (VAS) score. In the alpha frequency band, neural connectivity expressed as AEC was seen between the right SII and right precuneus, and between the right SII and the right insula (q < 0.01). In the theta frequency band, the AEC values between the right SII and right PCC correlated with the pain VAS score (q < 0.01). Abbreviations: SII, secondary somatosensory cortex; PCC, posterior cingulate cortex; VAS, visual analogue scale. Solid lines regression curves.

The AEC and VAS values were significantly correlated for the SII and the precuneus (q < 0.05, FDR) and for the SII and insular cortex (q < 0.05, FDR) in the alpha frequency band in the right hemisphere. In the theta frequency band, the AEC and VAS values were correlated for the SII and PCC in the right hemisphere (q < 0.05, FDR). Correlations between the AEC and VAS values are shown in Fig. 5. In the beta frequency band, there were no such correlations with VAS scores.

### 3.5 Correlation between pain or neural connectivity and age

It is reported that the neural connectivity of the default mode network (DMN) is positively correlated with the aging process (Broyd et al., 2009). There was no correlation between age and VAS scores in participants of the present study. Regarding the neural connectivity among the pain-related cortical regions, there was no relationship between the values and participant age.

## 4 Discussion

The present study analyzed resting-state MEG signals obtained from patients with CRPS in order to elucidate brain activity reflecting chronic pain objectively. We found that neural activity expressed as cortical current density was less in participants with severe chronic pain than in those with no to mild chronic pain in the posterior-inferior temporal region and several pain-related cortices. Moreover, neural activity in the bilateral SI, SII, and precuneus, but not in the posterior-inferior temporal region, showed a negative correlation with subjective chronic pain. Subjective chronic pain, reflected as a VAS score, negatively correlated with neural connectivity, expressed as AEC, in the SII and three pain-related regions (the precuneus, insula, and PCC) in the right hemisphere.

### 4.1 Current density in temporal and occipital cortices

Participants with moderate to severe chronic pain showed less current density (neural activity) in the occipital, temporal, and pain-related cortices than those with no to mild chronic pain. Interestingly, there was no “hot-spot” of cortical activity in participants with moderate to severe chronic pain. Frequency analysis of the MEG signals showed that the power of the theta and alpha frequency bands was depressed in participants with moderate to severe chronic pain in those regions. Current density was depressed in participants with moderate to severe chronic pain, including depressed occipital-temporal alpha activity. Since alpha activity is present during awake and relaxed states (Berger, 1929, Goldman et al., 2002, Niedermeyer, 1987, Niedermeyer, 1997), participants with moderate to severe chronic pain might have been less relaxed during the measurement. However, areas showing differences in current density between groups involved not only occipital-temporal regions, but also areas outside of those regions (e.g. precuneus, and cingulate cortices) that form large-scale networks, such as the DMN (Greicius et al., 2003). This cortical distribution suggested that the DMN was affected in participants with moderate to severe chronic pain (Hsiao et al., 2017). In the theta frequency band, patients with fibromyalgia have been reported to show reduced connectivity around the temporal gyri and visual cortex as compared to that in patients with a longer pain history. Reduced theta frequency power around these regions in participants with moderate to severe chronic pain in the present study might be in line with this previous result (Choe et al., 2018). Therefore, resting-state cortical current density suggested that neural activity in the brain regions responsible for the DMN were affected by chronic pain, at least that at moderate to severe levels.

### 4.2 Cortical current density and neural connectivity

Current density in the SI, SII, and precuneus correlated linearly with subjective chronic pain, but not in the occipital-temporal and insula-ACC regions (Fig. 3, Fig. 4). The SI and SII cortices are known as the initial receptive cortical areas for pain stimulation and they have neural connections to pain-related cortices, according to previous electroencephalography and MEG studies (Perri et al., 2019, Peyron and Fauchon, 2019). Reduction of neural density and connectivity among pain-related cortices that show linear correlation with subjective chronic pain suggested that pain processing in these brain areas was functionally disrupted by chronic pain in patients with CRPS. Involvement of the precuneus in the reduction of current density and neural connectivity in participants with chronic pain also indicated changes in the DMN in patients with CRPS.

It may be important that a significant reduction in neural connectivity was observed in the right hemisphere, although there was a reduction in current density in both hemispheres. The right hemisphere is responsible for spatial attention and cognition (Joseph, 1988, Shulman et al., 2010). Disruption in attention and spatial or body recognition is reported in patients with chronic pain (Brun et al., 2019). The significant reduction in neural connectivity might have been related to right hemispheric-dominant functions, although we did not measure attention functions.

### 4.3 Possibility of secondary modulation of neural activity by chronic pain

Resting-state cortical activity includes not only neural activities primarily caused by chronic pain, but also neural activities related to physical and mental conditions at the time of the measurement. Chronic pain-related psychological reactions such as fatigue, irritation, or uncomfortableness might cause neural activities and connectivity among sites responsible for such emotional reactions via top-down mechanisms (Arnau et al., 2017, Bushnell et al., 2013, Chen and Heinricher, 2019, Malfliet et al., 2017, Perri et al., 2019). It is difficult to distinguish primary pain-related cortical activity from that of secondary activity modulated by chronic pain that may have been sustained for months to years in patients with CRPS. We speculate that the pattern of current density or neural connectivity shown was the consequence of a balance of subjective pain processing and top-down modulation of pain sensation, which might be secondary brain responses caused by the primary level of chronic pain.

### 4.4 Limitations

This study has some limitations. The number of patients with CRPS was small, although statistical significance of differences was obtained. As previously discussed, in targeting the primary effects of CRPS, we cannot dismiss the likelihood that our results are an amalgam including secondary effects of chronic pain. Since we focused on the relationship between chronic pain and brain activity in patients with CRPS, further studies are needed to clarify whether the present findings could extend to patients with other types of chronic pain (Wakaizumi et al., 2019). We did not control for drug effects. One patient took fentanyl, two patients took tramadol, seven patients took pregabalin, and one patient took amitriptyline. One major reason for this was that discontinuation of medication could have caused physical and mental distress in patients. Another was that, had we attempted to suspend a course of treatment, the residual effects of drugs such as neuroleptics and analgesics taken by patients with CRPS might have lasted longer than a few days and it would have been difficult to achieve the desired washout. However, such drugs do not reduce neural activity or connectivity per se, although they may induce sleep.

### 4.5 Conclusions

We showed resting-state neural activity and connectivity between brain regions in patients with CRPS. Neural activities in the theta and alpha frequency bands were reduced in the occipito-temporal cortices and other brain regions responsible for pain processing in the DMN. Neural connectivity between the SII and some pain-related cortices (i.e. the precuneus, insula, and PCC) were reduced by chronic pain in CRPS. Disruption of pain processes and functions in the DMN was suggested in patients with CRPS. Our method targeting the neural mechanism of pain has the potential to offer a clinically objective means of evaluating it. In the near future, we may predict pain VAS scores by calculating connectivity or use these results to evaluate interventions. If secondary changes in brain activity make chronic pain refractory, brain activity measurement may indicate the optimal therapeutic approach.

## Funding

This research was supported by the 10.13039/100009619Japan Agency for Medical Research and Development (AMED) (grant number 17ek0610009h0003) and the 10.13039/501100001691Japan Society for the Promotion of Science (JSPS) (Grant-in-Aid for Scientific Research [KAKENHI] grant number 19K09647).

## CRediT authorship contribution statement

**K. Iwatsuki:** Conceptualization, Funding acquisition, Investigation, Project administration, Writing - original draft, Writing - review & editing. **M. Hoshiyama:** Conceptualization, Formal analysis, Funding acquisition, Investigation, Methodology, Writing - original draft, Writing - review & editing. **A. Yoshida:** Investigation. **J. Uemura:** Formal analysis. **A. Hoshino:** Formal analysis. **I. Morikawa:** Formal analysis. **Y. Nakagawa:** Investigation. **H. Hirata:** Conceptualization, Funding acquisition, Methodology, Supervision.
